# The Authenticity Scale: Validation in Russian Culture

**DOI:** 10.3389/fpsyg.2020.609617

**Published:** 2021-01-13

**Authors:** Sofya Nartova-Bochaver, Sofia Reznichenko, John Maltby

**Affiliations:** ^1^School of Psychology, National Research University Higher School of Economics, Moscow, Russia; ^2^College of Medicine, Biological Sciences and Psychology, University of Leicester, Leicester, United Kingdom

**Keywords:** Authenticity Scale, wellbeing, validation, reliability, Russian culture

## Abstract

The correlational study is aimed at validating the *Authenticity Scale* in Russian culture. Authenticity is considered a trait responsible for a person’s ability to be oneself. It helps people resist environment pressure and prevent self-alienation, which contributes to maintaining psychological wellbeing. The original Authenticity Scale includes three subscales: *Authentic Living, Accepting External Influence*, and *Self-Alienation*. In total, 2,188 respondents (*M*_age_ = 26.30, *SD*_age_ = 13.81; 78.1% female) participated in the survey. The dimensionality of the *Authenticity Scale* and its measurement invariance across sex, age, and depression rate subgroups was examined with exploratory and confirmatory factor analyses; the original tripartite structure was kept. Convergent validity was tested through correlation analyses with the *Warwick-Edinburgh Mental Well-being Scale*, the *International Positive and Negative Affect Schedule Short-Form*, the *Centre of Epidemiological Studies-Depression Scale*, the *Rosenberg Self-Esteem Scale*, and the *Satisfaction with Life Scale*. According to the CFA results, the structure of the Russian version differs from the original one slightly (item 1 was moved from the subscale *Authentic Living* to the subscale *Accepting External Influence* and item 4 was excluded); however, the modified factor model showed the best absolute and comparative fit statistics [CFI = 0.961, TLI = 0.949, RMSEA = 0.050 (90% CI [0.40; 0.60]) and SRMR = 0.037]. The reliability (McDonald’s Omega) of the *Authenticity Scale* subscales was satisfactory and ranged from 0.78 to 0.84. It was revealed that youth are more likely to have high scores on *Accepting External Influence* and *Self-Alienation* than adults. Men and women did not significantly differ on the sub-scores of *Authenticity Scale*. Multigroup CFA also showed that *Authenticity Scale* scores may be biased in people with high levels of clinical depression, in terms of the item intercepts. *Authentic Living* is positively connected with mental wellbeing, self-esteem, positive affect, satisfaction with life, and negatively with depressive symptoms and negative affect; reverse trends were found for *Accepting External Influence* and *Self-Alienation* subscales. The Russian version of the *Authenticity Scale* is a valid, reliable tool that may be recommended for use in various areas of non-clinical practice.

## Introduction

The current study validates the *Authenticity Scale* by Wood et al. (2008) in Russian culture.

Personal authenticity is one of the most demanded values in today’s hectic and changing society where people change their social, economic, and educational status and even identities several times throughout their lives. To be authentic, to be oneself – these intentions and wishes are essential for the psychological wellbeing in both eudemonic and hedonic meanings (Kernis and Goldman, 2006; Smallenbroek et al., 2017; Sutton, 2020).

Personal authenticity contributes to different beneficial psychological phenomena, such as positive affective relationships, subjective well-being, high self-esteem, compassion and self-compassion, and emotional regulation (Sheldon et al., 1997; Neff and Suizzo, 2006; Theran, 2010; Boucher, 2011; English and John, 2013; Kifer et al., 2013; Wickham, 2013; Bochaver et al., 2019; Zhang et al., 2019; Yanchenko and Nartova-Bochaver, 2020). In addition, it is a buffer between stress and its consequences or a negative predictor of psychological vulnerability (Goldman and Kernis, 2002; Satici et al., 2013). Therefore, this is a quality that is worth exploring, measuring, and developing.

In its most general form, authenticity can be defined as a person’s ability to be true to themselves, their nature, and their life trajectory. It seems to be one of the “troubling” challenging concepts, however, because it exists separately from any objective criteria and implies a high coherence with a specific person’s feelings or ideas that are not always explicit (Jones, 2010). This feature of authenticity makes it difficult to study the content validity of the corresponding tools, and, for this reason, most validation studies are limited to the examining divergent validity only. Moreover, understanding authenticity differs across cultures, and for cross-cultural study, a stable and reliable instrument capturing the core content of authenticity is needed.

The term “authenticity” is of Greek origin (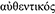 means true, genuine, and 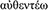 means to be full of energy). In Russia, the ability to be oneself has traditionally been understood as respect for one’s social class, origin, and economic status, and the preservation of honor and dignity even at the cost of one’s own life. In the Soviet Union, where people experienced intense social pressure that resulted in various forms of self-alienation (renouncing their ancestors, alcoholism, doublethink), personal authenticity became an exaggerated value, because it was the only way to preserve oneself as a person. Authenticity was expressed in the ability to defend their worldview, no matter what, and resist social pressure. At that time, the core of authenticity was personal independence and the absence of social conformity (Chudnovsky, 1981).

Contemporary psychology considers personal authenticity from both interpersonal and intrapersonal perspectives (Grégoir et al., 2014); the authentic person follows both their individuality and their life context and course. Several manifestations of personal authenticity are identified. According to Harter, it involves “owning one’s personal experiences, be they thoughts, emotions, needs, wants, preferences, or beliefs, processes captured by the injunction to ‘know oneself”’ (Harter, 2002, p. 382). Sheldon et al. (1997) considered authenticity a sign of a person’s self-organization. Kernis and Goldman (2006), based on the existential paradigm, operationalize authentic functioning from four perspectives: self-understanding, recognizing their ontological realities objectively, actions, and the features of interpersonal relationships. In the humanistic person-centered conception, the authenticity phenomenon assumes consistency between a person’s primary experience, their conscious or symbolized awareness, and their real communication and behavior (Barrett-Lennard, 1998). Discrepancy between experience and awareness results in self-alienation; coherence between awareness and behavior leads to authentic living. Finally, if a person is inclined to perceive influences from outside too strongly, this can increase self-alienation and weaken the ability to live authentically. All of definitions listed above emphasize the internal consistency of the individual in the first line, the correspondence of their desires, opinions, roles, decisions, and behavior. Finally, the definition of authenticity as harmony between oneself and one’s actual course, including destiny, by Russian scientists differs somewhat from this understanding (Nartova-Bochaver, 2011; Leontiev and Shilmanskaya, 2019; Nartova-Bochaver et al., 2020).

Authenticity has been studied not only as a trait but also as a fluent state (Fleeson and Wilt, 2010; Pinto et al., 2011; Lenton et al., 2016), “a sense of authenticity” (Ito and Kodama, 2007) or a contextual phenomenon (Robinson et al., 2013; Van den Bosch and Taris, 2014). To sum up, personal authenticity remains a rather vague concept and, as Kernis and Goldman (2006) noted, at times, seemed not to be easy to describe due to language limitations while the opposite pole, self-alienation, or false self, has been widely investigated. Thus, no matter how different manifestations of authenticity are studied – as states or features of relationships – there is always a trait authenticity behind them, which, again, makes it very important to have an appropriate tool for measuring personal authenticity.

Many attempts have been made to create a reliable tool for studying and measuring authenticity. The easiest ones were direct items or questions, for instance, “I have freely chosen this way of being” (Sheldon et al., 1997), “How much were you acting like your true self?” (Fleeson and Wilt, 2010), or “I can be myself with others” (Kraus et al., 2011). As authenticity research expands, more accurate standard tools are needed. One of the first measures, *Authenticity Inventory* (Kernis and Goldman, 2006) including 45 items and four subscales, represented an existential understanding of authenticity very accurately and predicted many variables of positive functioning (Kernis and Goldman, 2006; Davis et al., 2015). Its disadvantages are the length and unstable structure (White, 2011; Lenton et al., 2016). That is why the short tripartite *Authenticity Scale* by Wood et al. (2008) has become more popular among authenticity researchers. Moreover, a special modification of this scale has been developed for the work context – it measures how authentic a person feels when performing their professional duties (Van den Bosch and Taris, 2014; de Carvalho Chinelato et al., 2015).

*Authenticity Scale* (Wood et al., 2008) was developed in line with Rogers’s person-centered approach and based on Barrett-Lennard’s tripartite model of authenticity (Barrett-Lennard, 1998). Considering a trait, personal authenticity includes three components: *Authentic Living* means to be consistent with one’s beliefs, actual feelings, and objective reality; not *Accepting External Influence* when it goes against personal beliefs; and a lack of *Self-Alienation*. *Authentic Living* contributes to the *Authenticity Scale* positively whereas *Accepting External Influence* and *Self-Alienation* negatively, hence, in the original version, most items describe rather the absence of authenticity, and this tendency has become even stronger in the Russian modification of the tool. To our knowledge there have not been any adaptations of tools measuring personal authenticity in Russia.

Adaptations of the *Authenticity Scale* have been made in Canada, Italy, Iran, Portugal, Russia, Sweden, Serbia, and Turkey (Shamsi et al., 2012; İlhan and Özdemir, 2013; Di Fabio, 2014; Grégoir et al., 2014; Vainio and Daukantaitė, 2015; Grijak, 2017; Balbini et al., 2018). In all countries the original three-factor structure was kept, with the exception of Serbia, where a bi-factor structure was obtained (Grijak, 2017); psychometric properties (consistency and test–retest reliability) were satisfactory. To study the convergent validity, which turned out to be high everywhere, researchers used the following indicators: subjective wellbeing and autonomy (İlhan and Özdemir, 2013; Grégoir et al., 2014), self-esteem, satisfaction with life, and positive/negative affect (Di Fabio, 2014; Grégoir et al., 2014; Grijak, 2017; Vainio and Daukantaitė, 2015); a sense of coherence and harmony in life (Vainio and Daukantaitė, 2015). In each case, it was shown that the scale really distinguished respondents and could be used to solve research problems.

Although some studies found sex differences in the context of relational authenticity (Harter et al., 1998; Smolak and Munstertieger, 2002; Theran, 2011), there is very little evidence for sex differences in dispositional authenticity, beginning from the first original version of the *Authenticity Scale* (Wood et al., 2008). Satici et al. (2013) did not find any sex differences in *Authenticity Scale* scores. Wenzel and Lucas-Thompson (2012) also found no significant differences in trait authenticity based on sex. Bardadymov (2012) found that in male teenagers *Accepting External Influence* is higher compared to females. As for the age trends, the data is scarce as most studies were conducted on the one age group only – students or teenagers. However, Rogers (1961) believed that people are authentic only at an early age, and that authenticity is lost because of the acceptance of influences from outside. In the absence of reliable and stable data regarding age trends and sex differences in personal authenticity, we do not propose any hypotheses concerning age and sex.

The current study follows the same way of the validation of the *Authenticity Scale* in Russia as in previous research. Based on the results of construct validity testing observed in the original validation study (Wood et al., 2008) and following the conceptualization of the *Authenticity* as an attribute of a psychologically healthy person (Sheldon et al., 1997; Kernis and Goldman, 2006; Lopez and Rice, 2006; Blomgren and Strååht, 2018) we predicted positive connections of the *Authenticity Scale* and indicators of mental wellbeing and negative – with indicators of ill-being.

We put forward the following hypotheses:

H1: The Russian version of the *Authenticity Scale* will keep the same three-factor structure as the original version.

H2a: *Authentic Living* will be positively connected with other parameters of wellbeing (mental wellbeing, self-esteem, positive affect, and satisfaction with life), whereas *Accepting External Influence* and *Self-Alienation* will be negatively connected with them.

H2b: *Authentic Living* will be negatively connected with markers of ill-being (depressive symptoms and negative affect), whereas *Accepting External Influence* and *Self-Alienation* will be positively connected with them.

## Materials and Methods

### Participants

The total sample included 2,188 participants aged 18–70 years (*Me*_age_ = 20, *M*_age_ = 26.30, *SD*_age_ = 13.81; 78% females); not all respondents completed all measures. Most participants were students of Russian universities (*n* = 1,227; 56%) or people who had higher education (*n* = 513; 24%), taken advanced training in psychology, or participated in open elective courses in psychology; 448 (20%) respondents did not provide information on their level of education. Participants were included in the sample if the following criteria were met: (a) age ≥ 18; (b) written informed consent was given; (c) willingness to comply with all study-related treatments. Exclusion criteria were: (à) Russian is not a native language of the participant; (b) duration of residence in Russia less than 5 years. Data were collected in 2014–2018 from students in class as a part of their homework using the pencil-and-paper technique or via the on-line service 1ka.si; it took about 35 min in total, and participants could interrupt the survey if needed. To get more data from a broader spectrum of the population, we used the snowball technique. In addition to the main questionnaires, we asked respondents to provide demographic information (age, sex, level of education, citizenship, and length of residence in Russia). Participation was voluntary; students were given extra credit for completing the questionnaires but no any remuneration. The study was conducted in accordance with the Declaration of Helsinki and approved by the Commission for the Ethical Evaluation of Empirical Research Projects of the Department of Psychology of the National Research University Higher School of Economics (report of the Ethical Commission meeting number 8 of September 1, 2020). All of respondents provided their written informed consents to participate in this study and to publish the results anonymously.

### Measurement Instruments

#### Authenticity Scale

The *Authenticity Scale* measures an individual’s self-reported sense of authenticity that comprises three aspects: *Authentic Living, Accepting External Influence*, and *Self-Alienation* (Wood et al., 2008). *Authenticity Scale* consists of 12 items describing each of three aspects, with which participants expressed their agreement on a seven-point Likert scale ranging from 1 (does not describe me at all) to 7 (describes me very well).

The original *Authenticity Scale* items were translated into Russian separately by Bardadymov (2012) and Robinson et al. (2013), who received permission to translate the tool into Russian. All wordings were discussed with professional linguists, edited to get the clearest version, and back-translated by a psychologist who had been working at a United Kingdom university for 6 years.

Unfortunately, the initial pilot study conducted on a sample of 112 people revealed problems with *Authentic Living* items: central tendency measures had a strong shift toward maximum values which seemed to be caused by the very high importance of authenticity in Russians’ lives so that they could not admit to being inauthentic (Table 1). The overall mean and median values for *Authentic Living* scale were much higher (*M* = 23.24, *Me* = 24) than those for *Accepting External Influence* (*M* = 15.41, *Me* = 15) and *Self-Alienation* (*M* = 12.11, *Me* = 11), while its standard deviation was inversely lower (*SD* = 3.54, 4.46, 5.62, respectively). This result was repeated in the two additional pilot studies on different samples (Bardadymov, 2012). Furthermore, the few articles providing descriptive statistics also demonstrated a negatively skewed distribution on *Authentic Living* scores, and skewed to the right *Accepting External Influence* scores (Grijak, 2017), as well as much higher mean and median values of *Authentic Living* compared to other subscales (Grégoir et al., 2014; Balbini et al., 2018). Considering these trends, the authors concluded that the items of *Authentic Living* subscale exposed a social desirability bias and, thus, needed editing.

**TABLE 1:** Descriptive statistics of the *Authenticity Scale* subscales reported in previous studies and in the current study.

	*N*	Mean	*SE*	*Me*	Min	Max	*SD*	Skewness	Kurtosis	The *Authenticity Scale* version
*AL*	112	23.2	–	24.0	6.0	28.0	3.5	–1.7	5.2	Bardadymov, 2012: originally
*AEI*		15.4	–	15.0	6.0	27.0	4.5	0.3	–0.3	worded items in the *AL* subscale,
*SA*		12.1	–	11.0	4.0	25.0	5.6	0.5	–0.6	original factor structure
*AL*	2188	18.35	0.11	19.0	4.0	28.0	5.12	–0.23	–0.47	Current study: *AL* subscale
*AEI*		15.21	0.10	15.0	4.0	28.0	4.94	–0.08	–0.50	with negatively reworded items,
*SA*		12.83	0.13	12.0	4.0	28.0	6.11	0.39	–0.74	original factor structure
*AL*	2188	13.49	0.09	14.0	3.0	21.0	4.24	–0.20	–0.63	Current study: *AL* subscale
*AEI*		13.64	0.11	13.0	4.0	28.0	5.19	0.18	–0.61	with negatively reworded items,
*SA*		12.56	0.11	12.0	4.0	28.0	6.10	0.33	–0.29	final factor structure, resulted in CFA

*AL, *Authentic Living*; AEI, *Accepting External Influence*; SA, *Self-Alienation*.*

To reduce respondents’ tendency to give overly positive self-descriptions about their authentic living, four items (1, 8, 9, 11) from *Authentic Living* were negatively reworded (see Appendix). Thus, the item 1 “I think it is better to be yourself than to be popular” was rephrased as “I think it is better to be popular than to be yourself,” the item 8 “I always stand by what I believe in” – as “I do not always succeed in upholding what I believe in,” the item 9 “I am true to myself in most situations” as “I can’t say that I am true to myself in most situations,” and the item 11 “I live in accordance with my values and beliefs” as “I don’t live in accordance with my values and beliefs.” Table 1 reflects the changes in the descriptive statistics of the authenticity subscales before the reformulation of the statements (Bardadymov, 2012), as well as after the statements in the *Authentic Living* subscale were negatively reworded. The changes in skewness and kurtosis measures show that negatively reworded items allowed us to normalize the values on the *Authentic Living* subscale, so in this research, we used the modified version of the *Authentic Living*.

By studying the convergent validity of the *Authenticity Scale*, we tried to replicate the procedure for examining the scale in other cultures, using the familiarity of the constructs of the personal authenticity and psychological wellbeing (İlhan and Özdemir, 2013; Di Fabio, 2014; Grégoir et al., 2014; Vainio and Daukantaitė, 2015; Grijak, 2017). Unfortunately, at the moment there is no other valid tool in Russia that would measure trait authenticity. Six additional measures, reflecting common mental wellbeing concepts and emotional state, were included in this study to examine convergent validity. All of them have already been adapted for Russian culture.

#### Mental Wellbeing

The *Warwick-Edinburgh Mental Well-Being Scale (WEMWBS)* was used to measure an individual’s self-reported mental wellbeing during the last 2 weeks (Tennant et al., 2007; Russian version: Robinson et al., 2013). It is a unidimensional scale consisting of 14 items regarding positive mental health. Respondents answer using a five-point scale ranging from 1 to 5. *WEMWBS* is a commonly used measure of wellbeing, which is stable across cultures for reliability, construct, and convergent validity. Example of items: “I’ve been feeling cheerful.” In the current study, Cronbach’s alpha was 0.89.

#### Subjective Wellbeing

Following the developers of the original version of the scale, we measured subjective wellbeing using a set of methods and interpreted it as including high satisfaction with life and positive affect and low negative affect.

We used the *Satisfaction with Life Scale (SWLS)* reflecting cognitive-judgmental aspects of quality of life (Diener et al., 1985; Russian version: Osin and Leontjev, 2008). It is a seven-point scale, including five items (e.g., “So far, I have gotten the important things I want in life”). *SWLS* is the most commonly used measure of the evaluative component of subjective wellbeing and has a high degree of temporal stability. In the current study, alpha was 0.83.

*International Positive and Negative Affects Schedule Short-Form (PANAS-SF)* was developed by Thompson (2007) as a culturally independent measure of the affective component of subjective wellbeing and includes only 10 items. These 10 items are derived from the original version of the PANAS (Watson et al., 1988), consisting 20 items. The five positive affective states are: active, determined, attentive, inspired, and alert. The five negative affective states are: afraid, nervous, upset, hostile, and ashamed. Respondents are asked to rate the items on a 5-point scale (“not at all” to “extremely”) according to the extent to which each describes the way they have felt during past few weeks (Thompson, 2007; Russian version: Osin, 2012). In this study, we had to remove item 3 (“Alert”) from Positive Affect since it damaged the subscale’s internal consistency. The removal of this item increased Cronbach’s alpha from 0.44 to 0.67. Although reliability of the reduced four-item Positive Affect is marginally below the accepted alpha values, it is still acceptable for the adapted psychometric instrument with few items and just for research (non-clinical) purposes (Taber, 2016). Negative Affect and overall *PANAS-SF* alphas were 0.80 and 0.75, respectively.

#### Self-Esteem

The *Rosenberg Self-Esteem Scale (Self-Esteem)* was chosen to measure an individual’s overall subjective emotional evaluation of oneself, such as despair or pride (Rosenberg, 1965; Russian version: Prihozhan, 2002). It is a 10-item scale with responses on a five-point scale. Example of items: “On the whole, I am satisfied with myself.” The Cronbach’s alpha in our study was 0.83.

#### Depression

The *Centre of Epidemiological Studies-Depression Scale (CES-D)* was used to measure the severity of depressive symptoms. The scale reflects individuals’ self-reported personal state in the past week (Radloff, 1977; Russian version: Andryshchenko et al., 2003). It includes 20 items assessed by a four-point scale (e.g., “I thought my life had been a failure.”). In the current study, Cronbach’s alpha was 0.89.

### Analytic Strategy

To study the factor structure and construct validity of the *Authenticity Scale*, the total sample (*n* = 2,188) was split randomly into three subsamples: (1) EFA (33.3% observations, *n* = 730); (2) CFA1 (33.3%, *n* = 729), and CFA2 (33.3%, *n* = 729). In the EFA subsample, EFA was carried out; CFA and Structural Equation Modeling (SEM) were applied to the CFA1 subsample. Then, we enhanced the cross-validity of the optimal CFA model using the CFA2 subsample. The entire sample was used in a multi-group CFA to evaluate measurement invariance across sex, age, and depression rate, to test internal and construct reliability, and to estimate the relationships of the *Authenticity* subscales with other constructs.

To identify the number of factors to extract before EFA Horn’s parallel analysis (both principal axis analysis and principal components) was conducted via psych package in R using minimum residual factor method. The factors’ eigenvalues of the observed data were compared with the eigenvalues for a principal components and principal axis factor analysis from 1000 randomly generated correlation matrices. Both EFA and CFA were performed with the robust ML (MLR) rescaling-based estimator due to its ability to handle ordinal variables and non-normal data. Overall ten factor models (see section “Confirmatory Factor Analysis”) of the *Authenticity Scale* were tested with CFA. To test how well each of them fits into observed values a set of commonly used goodness-of-fit indices were used: the Comparative Fit Index (CFI), the Tucker Lewis Index (TLI), the Root Mean Square Error of Approximation (RMSEA), *p*-value of Close Fit (PCLOSE), and the Standardized Root Mean Square Residual (SRMR). Both CFI and TLI values exceeding 0.95 indicate good model fit (Hu and Bentler, 1999; Kline, 2016). RMSEA values smaller than 0.06 indicate close fit, and values smaller than 0.08 are considered mediocre (MacCallum et al., 1996; Hu and Bentler, 1999). SRMR value smaller than 0.08 (Hu and Bentler, 1999; Kline, 2016) indicate an acceptable fit. The models’ parsimony was estimated with the Akaike’s Information Criterion (AIC) and the Expected Cross-Validation Index (ECVI): the model with the smallest value of AIC or ECVI was considered best (Akaike, 1987; Browne and Cudeck, 1992).

The best fitted factor model’s measurement equivalency across sex, age, and depression rate subsamples was conducted via Multigroup CFA. Measurement invariance testing in each subsample contained three assessments with increasing constraints: baseline configural model (no constraints), metric model (factor loadings of the first order and general factors constrained), and scalar model (constrained factor loadings and intercepts of the first order and general factors). The CFA model was tested separately for each group and all groups together. Model fit was evaluated by CFI, TLI, RMSEA, SRMR, and PCLOSE. Evaluation of the invariance was conducted by the assessment of changes in the fit index. The invariance criteria used were ΔCFI and ΔTLI less than 0.01, ΔRMSEA less than 0.015, and ΔSRMR less than 0.03 (Chen, 2007). The magnitude of latent mean structure difference was specified using the Hedge’s *g* statistic, which provides a measure of effect size (ES) weighted according to each sample’s relative size. Hedge’s g has the same rule of thumb for interpretation, as suggested by Cohen (1988): *g* = 0.2 is a small effect, *g* = 0.5 is a medium effect, and *g* = 0.8 or above for a significant effect.

The internal reliability of the *Authenticity Scale* was estimated for the entire sample with the McDonald’s omega (McDonald, 1999). Omega is considered to be a more accurate method of measuring reliability than the traditionally used Cronbach’s alpha, because omega does not require uncorrelated error variances and gives the proportion of variance in scale scores accounted for by a general factor, usually from second-order factor analysis, while Cronbach’s alpha tends to overestimate reliability when the error variances of the indicators are correlated and can be underestimated when the number of items is small. The commonly used cutoff value for McDonalds’ omega is 0.7 (Hair et al., 2010). However, since all previous validation studies of the *Authenticity Scale* used Cronbach’s alpha, we also felt it necessary to provide alpha values. This will allow researchers to compare the reliability of the scale validated in different countries. The threshold of 0.7 for Cronbach’s alpha indicates acceptable reliability (Taber, 2016).

Convergent validity was investigated by examining correlations between the *Authenticity Scale* subdomain scores and several associated measures, namely *WEMWBS, SWLS, PANAS-SF, Self-Esteem*, and *CES-D* in the context of a power analysis. According Cohen’s (1988) recommendations, Pearson *r* values of 0.10–0.29, 0.30–0.49, and 0.50 and higher were used to demarcate small, medium, and large size effects, respectively.

For performing CFA and SEM analyses, we used the packages psych 1.9.12.31 (Revelle, 2020), lavaan 0.6-6 (Rosseel, 2012), and semTools (Jorgensen et al., 2020) implemented in the R Software and Programming environment 4.0.2 (R Core Team., 2020). We used SPSS v. 23, GPower v. 3.1, and MS Excel 2016 software for other analyses.

## Results

### Missing Value Analysis and Testing Statistical Assumptions

Initial sample data (*N* = 2,256) had 42 (1.9%) missing values for the *Authenticity Scale* variable; all these cases were deleted with listwise deletion. For testing outliers in the entire sample, the Mahalanobis Distance Test was performed. We have kept univariate outliers but removed multivariate non-normal observations [χ^2^(12) critical value = 32.91, *p* < 0.001] from the samples. Overall, 26 (1.2%) cases including missing values or outliers were removed from the initial sample (*N* = 2,256). Thus, the total sample included 2,188 observations.

**FIGURE 1:**
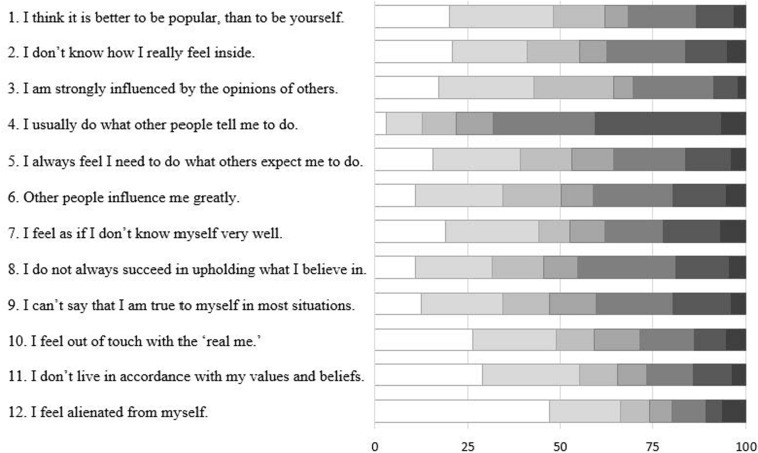
Distribution of responses for the total sample (*N* = 2188).

To measure multivariate normality in four samples (Total sample, EFA, CFA1, and CFA2 subsamples), we conduct Mardia’s multivariate kurtosis and skewness tests. The data in all four samples violated the normality assumption according to statistically significant Mardia’s Skewness test (*p* < 0.01). Only Mardia’s Kurtosis showed multivariate normality (*p* > 0.1). Similar results were revealed while estimating univariate normality with the Shapiro–Wilk test: The null hypothesis was rejected in all samples (*p*-values < 0.001) for each item, except 2, 6, 7, and 10, indicating a univariate normality deviation. However, the absolute values of skewness (sk) and kurtosis (ku) for each item computed for the entire sample did not determine substantial non-normality, but demonstrated slightly platykurtic distributions of all items and a right-skewed distribution of the item 12: item 1 (sk = 0.46; ku = −1.08); item 2 (sk = 0.24; ku = −1.24); item 3 (sk = 0.43; ku = −0.94); item 4 (sk = −0.79; ku = −0.29); item 5 (sk = 0.22; ku = −1.18); item 6 (sk = 0.13; ku = −1.23), item 7 (sk = 0.25; ku = −1.35), item 8 (sk = −0.04; ku = −1.23), item 9 (sk = 0.04; ku = −1.25), item 10 (sk = 0.48; ku = −1.02), item 11 (sk = 0.64; ku = −0.92), item 12 (sk = 1.11; ku = −0.07). This outcome is consistent with the results shown in Figure 1, where most items demonstrate a bias toward lower scores (especially in 11 and 12), with a more symmetrical distribution in 6 and 7, and an increased bias and potential ceiling effect in 4.

We had no bivariate correlations higher than 0.90, suggesting that the additivity assumption was met. The linearity assumption was tested with QQ-plot, comparing the sample quantiles to the theoretical quantiles of the posited distribution, and this was also met. The scatterplot of standardized fitted values (predicted score for each person), predicting standardized residuals, showed that the variance of the residuals was constant across the full range of fitted values, thus, supporting the homogeneity and homoscedasticity assumptions. We recode the reversed (negatively worded) items (1, 8, 9, and 11) before starting the EFA.

### Exploratory Factor Analysis

The data of the EFA subsample was used to perform the EFA of the *Authenticity Scale*. The scree-plot demonstrated a three-factor structure (with eigenvalues > 1.0), and together these factors accounted for 57.14% of the variance (*Self-Alienation* factor – 35.15%; eigenvalue 4.22; *Accepting External Influence* factor –13.61%; eigenvalue 1.63; *Authentic Living* factor – 8.39%; eigenvalue 1.01). The parallel analysis revealed that there are three factors and only two components in the dataset (see Figure 1). Three-factor solution better fitted empirical data than two-factor structure did according to SRMR (0.04 vs. 0.05), TLI (0.913 vs. 0.887), and RMSEA (0.069 vs. 0.076). With regard to these results, the theoretical model, and previous studies confirming the presence of three subscales in the *Authenticity Scale*, a three-factor solution was finally chosen for further analysis. The results of the EFA also showed that the three subscales were moderately intercorrelated. *Authentic Living* negatively correlated with *Self-Alienation* (*r* = −0.32, *p* < 0.001) and *Accepting External Influence* (*r* = −0.25, *p* < 0.001). *Accepting External Influence* positively correlated with *Self-Alienation* (*r* = 0.40, *p* < 0.001). These correlations are comparable to those obtained by Wood et al. (2008), ranging from −0.44 to 0.40.

As these factors were theoretically related and empirically moderately correlated, they were then extracted using oblimin rotation. CFI (0.952) and SRMR (0.04) reached the recommended level of fit. RMSEA [0.069 (90% CI [0.06; 0.08])] indicated mediocre fit (MacCallum et al., 1996), while TLI (0.913) remained below acceptable range. We found it possible to proceed specification of this three-factor model via CFA, taking into account a caution against being strictly dependent on cutoff when considering unspecified models during EFA (Hayduk et al., 2007).

No cross-correlations above 0.30 were found. Table 2 shows the initial and extracted communalities and standardized factor loadings (pattern matrix) based upon the correlation matrix for all 12 items. In general, the composition of the items repeats the original structure of the *Authenticity Scale* proposed by Wood et al. (2008), with the only exception that item 1 (one of those that was negatively worded) has gone into *Accepting External Influence* subscale instead of *Authentic Living*. In the next research stage, we examined the *Authenticity Scale* factor structure with CFA.

**TABLE 2:** Communalities and factor loadings from exploratory factor analysis with oblique rotation.

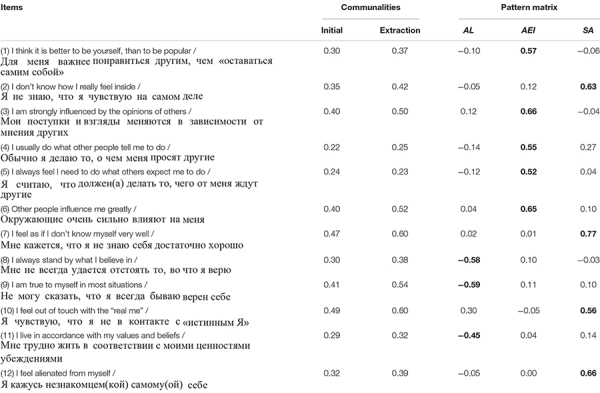

*AL, *Authentic Living*; AEI, *Accepting External Influence*; SA, *Self-Alienation*. Highest loadings for each factor in bold type. The scores items 1, 8, 9, 11 were reversed before EFA.*

### Confirmatory Factor Analysis

Ten factor models were tested with CFA based on data provided by the CFA-1 subsample (see Table 3).

**TABLE 3:** CFA fit statistics for the tested models of the *Authenticity Scale.*

Model	χ^2^	df	CFI	TLI	RMSEA [95%-CI]	PCLOSE	SRMR	AIC	ECVI
Model 1 Unidimensional	564.47	54	0.760	0.706	0.114 [0.106–0.122]	0.000	0.087	33439.19	0.950
Model 2 three-factor correlated	276.10	51	0.894	0.863	0.078 [0.069–0.086]	0.000	0.061	33113.01	0.502
Model 3 three-factor correlated: item 1 in *AEI*	209.28	51	0.934	0.926	0.065 [0.057–0.074]	0.002	0.050	33037.79	0.399
Model 4 three-factor Higher-Order, original	276.10	51	0.894	0.863	0.078 [0.069–0.086]	0.000	0.061	33113.01	0.502
Model 5 three-factor Higher-Order, modified (item 1 in *AEI*)	209.28	51	0.934	0.926	0.065 [0.057–0.074]	0.002	0.050	33037.79	0.399
Model 6 three-factor Higher-Order, modified (item 1 in *AEI*, item 4 deleted)	122.19	41	0.961	0.949	0.050 [0.040–0.060]	0.347	0.037	30380.30	0.254
Model 7 three-factor Bifactor	179.20	42	0.935	0.899	0.067 [0.057–0.077]	0.002	0.047	33016.72	0.370
Model 8 three-factor Bifactor, i1 in *AEI*	145.31	42	0.953	0.925	0.058 [0.048–0.068]	0.082	0.036	32980.19	0.320
Model 10 two-factor Bifactor, i1 in *AEI*, item 4 deleted	156.67	45	0.947	0.923	0.058 [0.049–0.068]	0.069	0.037	32988.44	0.031

*AEI, *Acceptance External Influence* subscale.*

*The fit statistics of Model 9 is not shown in the table due to its non-convergence.*

In *Model 1*, a unidimensional factor structure with one general *Authenticity* factor was tested according to the maximum parsimony assumption. This model showed poor fit, suggesting the multidimensionality of the authenticity concept. Following the Portuguese version of the *Authenticity Scale* (Balbini et al., 2018), a three-correlated factor model (12 items, *Authentic Living, Accepting External Influence*, and *Self-Alienation* subscales are correlated) was tested in *Model 2*. The model was also rejected from further analysis due to poor fit indices. A three-correlated factor *Model 3* included a structure change based on modification indices (MI) analyses: item 1 *(“I think it is better to be popular than to be yourself”)* was moved from the *Authentic Living* to *Accepting External Influence*. The model showed better fit compared to *Model 2*, but the CFI, TLI, and RMSEA indices were still unacceptable. As the latent variables showed a high covariance with each other (from 0.60 to 0.75), we put a second set with the overall *Authenticity* factor in *Model 4* to explain that covariance. In this model, the *Authenticity Scale’s* original hierarchical structure was reproduced, including three uncorrelated first-order factors loaded highly on a higher-order *Authenticity* factor (Wood et al., 2008). The model fit values were not satisfactory, based on the CFI, TLI, and RMSEA. However, the three latent factors loaded highly on a higher-order authenticity factor, suggesting that hierarchical structure is more appropriate to explain relationships between latent variables than the correlated one. *Model 5* described a higher-order structure with the free orthogonal subscales; item 1 *(“I think it is better to be popular than to be yourself”)* was moved from the *Authentic Living* to *Accepting External Influence*. Like *Model 3*, this model had poor CFI, TLI, and RMSEA values. To make additional improvement of the model we checked other MI with values higher than 10 and the most significant values of the standardized expected parameter change (EPC). MI suggested that the model could be improved by drawing error covariances between the items 4 (*“I usually do what other people tell me to do”*) and 5 (*“I always feel I need to do what others expect me to do”*) (MI = 64.01; EPC = 0.24), and the items 4 and 10 (*“I feel out of touch with the ‘real me”’*) (MI = 12.55; EPC = 0.08), adding paths from the item 4 to the *Self-Alienation* (MI = 25.97; EPC = 0.17) and *Authentic Living* (MI = 15.77; EPC = 0.12) factors, and also a path between the item 10 and a higher-order *Authenticity* factor (MI = 13.82; EPC = 0.09).

**FIGURE 2:**
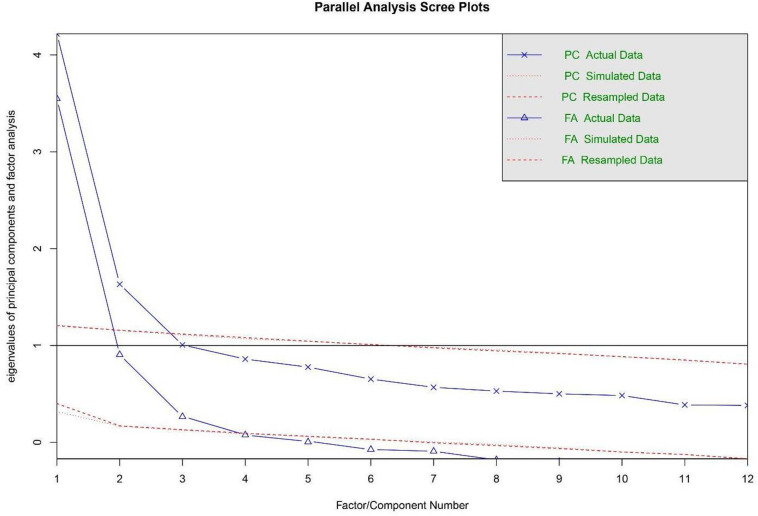
Parallel analysis. Principal component (PC) and factor analysis (FA) eigenvalues.

Since there was no strong theoretical rationale to add the error covariances between the items, we decided to remove item 4 from the model. There were two arguments for deletion of item 4: (a) this item had the lowest factor loading (0.50) compared to loadings of all other items; (b) item 4 correlated simultaneously with several latent factors. Thus, *Model 6* had a higher-order structure with item 1 as part of the *Accepting External Influence* sub-domain and excluded item 4 (see Figure 2). This model showed significantly better fit than *Model 5* (*p* < 0.001) and reached desired fit indices: CFI = 0.961, TLI = 0.949, RMSEA = 0.050 (90% CI [0.40; 0.60]) and SRMR = 0.037. *Model 7* was a bi-factor one with a general factor (*Authenticity*) and three specific orthogonal factors (*Authentic Living*, *Accepting External Influence*, and *Self-Alienation*) and included all 12 items without any structural modifications. This structure is based on the bi-factor CFA model proposed by Grijak (2017). All fit values, except CFI, TLI and RMSEA, were acceptable. To improve model fit, we moved item 1 to the *Accepting External Influence* factor and got an acceptable fit (*Model 8*), though it was poorer than in *Model 6*.

Nevertheless, when investigating the variances and squared multiple correlations of observed and latent variables, we found rather low *R*^2^ (0.294) of item 11 and also the positive but near-zero variance (0.281) of the *Authentic Living* factor. Referring to Hair et al. (2010), the items with *R*^2^ below 0.25–0.03 have to be considered candidates for removal. Only item 9 had a high factor loading on the *Authentic Living* subdomain while the other two items had not. When testing a bifactor *Model 9* with item 1 in the *Accepting External Influence* domain and item 4 rejected, we got a standardized regression weight more significant than 1 and negative variance for item 9, detecting a Heywood case. We assumed that the Heywood case in *Model 9* could be explained by over-extraction of latent factors in the previous structure solutions. So, we tested a bi-factor *Model 10*, with only two specific orthogonal factors (*Accepting External Influence* and *Self-Alienation)* and the general factor influencing all indicators. As in the *Model 9*, item 1 was moved in the *Accepting External Influence* domain and the item 4 was deleted. This model converged, but showed less satisfactory fit indices than *Model 6*.

**FIGURE 3:**
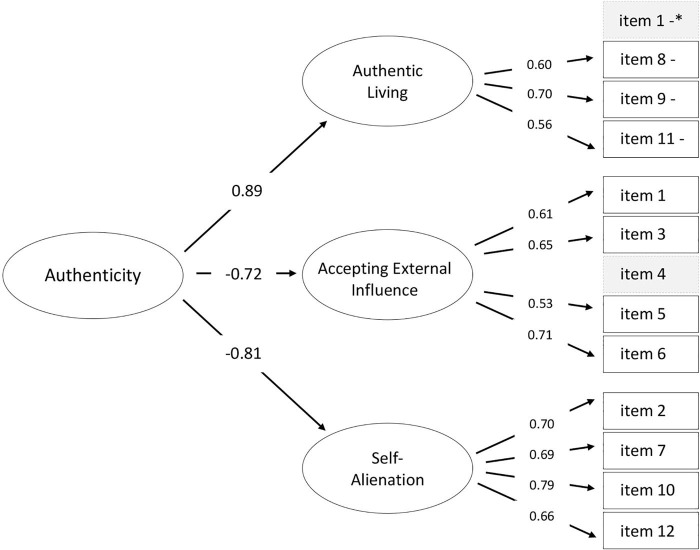
The optimal CFA model tested for the *Authenticity Scale* compared with the original model (Wood et al., 2008). Blocks dotted gray indicate excluded items. * – Item one was included in *Accepting External Influence.* “–” indicates negatively phrased items. Error variances omitted for clarity.

Therefore, we decided to return to the hierarchical *Model 6* as the most appropriate regarding its fit statistics and use it in further analysis. As shown in Figure 3, the hierarchical three-factor structure had the high loadings on the second-order authenticity factor, ranged from −0.72 (*Accepting External Influence*) to 0.89 (*Authentic Living*), as well as on first-order factors (between 0.53 and 0.79). Taken together, these factors explain 56.7% of the variance.

### Cross-Validation of the *Authenticity Scale* Factor Structure

After determining that the hierarchical three-factor model described trait authenticity best, as derived from the CFA1 subsample, cross-validation of the factor structure was performed. To test the replicability of the measurement results, we repeated CFA on another subsample (CFA2, *n* = 729) of the same size. Although cross-validation is insufficient requirement to protect against the sample idiosyncrasies, it is generally considered the preferred method of testing measurement stability of the scale (Kyriazos and Stalikas, 2018). All fit statistics of the replicated factor solution in the CFA2 subsample were acceptable [χ2 = 112.64, df = 41, CFI = 0.961, TLI = 0.949, RMSEA = 0.049 (90% CI [0.39; 0.59]) and SRMR = 0.036] and remained stable in comparison to fit measures of the CFA1 subsample (Byrne, 2011). The factor loadings of the cross-validated model were also comparable to the measures obtained in the CFA1 subsample: from 0.620 (*Accepting External Influence*) to 0.89 (*Authentic Living*), and between 0.491 and 0.802 for the observed variables.

### Measurement Invariance Across Sex, Age, and Depression Rate

To test the comparability of the *Authenticity Scale* values and compare the mean of latent variables across different groups, we examined measurement invariance across sex (males, *n* = 482 vs. females, *n* = 1,669), age (students, aged 17–25, *n* = 1,227 vs. adults, aged 26–73, *n* = 513), and depression rate (depressed-like, *n* = 228 vs. non-depressed, *n* = 985) subgroups (Table 4). The optimal cutoff for depression of 21 on the *CES-D* was used for optimizing true positive and false negative sample results (Henry et al., 2018).

**TABLE 4:** Measurement invariance fit statistics across sex, age, and depression rate.

Group	Type of MI	χ^2^	df	CFI	TLI	RMSEA	SRMR
Sex	(1) Configural	336.018	80	0.961	0.947	0.038	0.044
	(2) Metric	348.511	88	0.961	0.951	0.037	0.047
	**Δ 2-1**	**12**.**493**	**8**	**0**	**0.004**	−**0.001**	**0.003**
	(3) Scalar	384.968	99	0.957	0.952	0.036	0.047
	**Δ 3-2**	**36**.**457**	**11**	**−0.004**	**0.001**	**−0.001**	**0.001**
Age	(1) Configural	250.558	80	0.966	0.953	0.035	0.033
	(2) Metric	271.513	88	0.963	0.954	0.035	0.033
	**Δ 2-1**	**20**.**955**	**8**	**−0.003**	**0.001**	**0**	**0**
	(3) Scalar	462.572	99	0.918	0.927	0.046	0.033
	**Δ 3-2**	**191**.**059**	**11**	**−0.045**	**−0.027**	**0.011**	**0.000**
	(4) Partial*	315.184	96	0.953	0.945	0.044	0.035
	**Δ 4-2**	**43**.**671**	**8**	**−0.01**	**−0.009**	**0.009**	**0.002**
Depression rate	(1) Configural	250.659	80	0.954	0.937	0.042	0.040
	(2) Metric	268.462	88	0.952	0.941	0.041	0.041
	**Δ 2-1**	**17**,**803**	**8**	**−0**,**002**	**0**,**004**	**−0**,**001**	**0**,**001**
	(3) Scalar	457.133	99	0.904	0.894	0.055	0.047
	**Δ 3-2**	**188**,**671**	**11**	**−0**,**048**	**−0**,**047**	**0**,**014**	**0**,**006**
	(4) Partial**	399.387	96	0.920	0.909	0.050	0.043
	**Δ 2-1**	**130**,**925**	**8**	**−0**,**032**	**−0**,**032**	**0**,**009**	**0**,**002**

*Freed intercept of item 2, 5, and 12.

**Freed intercept of items 2, 11, and 12.

#### Sex

The configural hierarchical three-factor model contained insignificant variations in the male and female groups. The goodness-of-fit indices for the configural model indicated a close fit to the data in the male subsample (χ^2^ = 111,16, df = 40, CFI = 0.951, TLI = 0.933, RMSEA = 0.061, 95% CI [0.48; 0.74], PCLOSE = 0.088; SRMR = 0.041), and in the female subsample (χ^2^ = 218,51, df = 40, CFI = 0.965, TLI = 0.952, RMSEA = 0.052, 95% CI [0.45; 0.59], PCLOSE = 0.324; SRMR = 0.031). The configural model for all groups together also had an adequate fit to the data (see Table 4). In addition, all factor and item loadings in this model were large and highly significant (from 0.45 to 0.89, *p* < 0.01). The changes in the fit indices of the metric model indicated no significant decrement in fit from the configural model (ΔCFI = 0, ΔTLI = 0.004, ΔRMSEA = −0.001, ΔSRMR = 0.003). The scalar model had an adequate fit and had no significant difference in comparison to the metric model (ΔCFI = −0.004, ΔTLI = 0.001, ΔRMSEA = −0.001, ΔSRMR = 0.001). These results suggest that strong measurement invariance across sex is supported and the mean of latent variables in males and females can be compared. To test whether the latent mean of the *Authenticity* factors was invariant across sex, the means of latent variables in the female group were set to zero, whereas the latent means of male group were freely estimated. Males and females did not differ on levels of the *Authentic Living, Accepting External Influence*, and *Self-Alienation* factors (differences in means: −0.16, *p* = 0.453; 0.25, *p* = 0.420; 0.10, *p* = 0.706 respectively; Hedges’ *g*: 0.04, 0.04, 0.02 respectively).

#### Age

We grouped participants in youth (student) or adult groups using a cutoff age of 25 – the age when most Russian students complete their bachelor’s programs. The hierarchical three-factor model exhibited good model fit for students (χ^2^ = 169,41, df = 40, CFI = 0.964, TLI = 0.951, RMSEA = 0.051, 95% CI [0.44; 0.59], PCLOSE = 0.374; SRMR = 0.033) and excellent for adults (χ^2^ = 81.14, df = 40, CFI = 0.970, TLI = 0.959, RMSEA = 0.045, 95% CI [0.31; 0.59], PCLOSE = 0.713; SRMR = 0.035) by judging fit indices. All general factor and item loadings were significant (0.44–0.92, *p* < 0.01). The baseline multigroup model without any constraints also demonstrated very good fit. Constraining all factor loadings to be invariant across age samples resulted in a non-significant change in model fit as compared to the baseline model: ΔCFI = −0.003, ΔTLI = 0.001, ΔRMSEA = 0, ΔSRMR = 0 (see Table 4). Constraining all intercepts to be invariant across age groups resulted in a significant worsening of model fit as compared with the metric invariance model: ΔCFI = −0.045, ΔTLI = −0.027, ΔRMSEA = 0.11, ΔSRMR = 0. Based on the modification index, the intercepts of the items 2, 5, and 12 were freed for testing partial invariance. The intercept partially invariant model showed a non-significant difference in fit: ΔCFI = −0.01, ΔTLI = 0.009, ΔRMSEA = 0.009, ΔSRMR = 0.002. The means of latent variables in the student group were set to zero. The means of the *Authentic Living* did not differ in the student and adult subsamples (differences in means: 0.08, *p* = 0.704; Hedges’ *g* = 0.02), while *Accepting External Influence* and *Self-Alienation* means had significant differences (differences in means: −0.84, *p* = 0.000 and −1.41, *p* = 0.000; Hedges’ *g* = 0.20 and 0.42 respectively) indicating that students are more likely to accept external influences, when it goes against personal beliefs and to have an inconsistent sense of identity.

#### Depression Rate

The baseline hierarchical three-factor model were slightly misfitted for the depressed-like sample in terms of RMSEA (χ^2^ = 186.88, df = 40, CFI = 0.952, TLI = 0.932, RMSEA = 0.061, 95% CI [0.52; 0.70], PCLOSE = 0.445; SRMR = 0.040) and had a great fit for the non-depressed sample (χ^2^ = 81.44, df = 40, CFI = 0.963, TLI = 0.951, RMSEA = 0.047, 95% CI [0.32; 0.61], PCLOSE = 0.623; SRMR = 0.019). The baseline model for all groups together also had an adequate fit to the data (see Table 4). All general factor and item loadings were significant (0.48–0.96, *p* < 0.01). The metric invariance model difference test suggested that there is no significant deterioration in the model fit compared to the configural one (ΔCFI = −0.002, ΔTLI = 0.004, ΔRMSEA = −0.001, ΔSRMR = 0.011). But when comparing the scalar and metric invariance models, ΔCFI and ΔTLI significantly exceeded their cutoffs (ΔCFI = −0.048, ΔTLI = −0.047, ΔRMSEA = 0.014, ΔSRMR = 0.006). The partial scalar model with freed intercepts of the items 2, 11, and 12 also indicated decrement in model fit in comparison to the metric model in terms of both ΔCFI and ΔTLI. As partial scalar invariance was not supported, we can conclude that the item intercepts are not similar for people of different levels of depression and therefore there is no evidence to guarantee that mean comparisons of the *Authenticity Scale* across the depression rate samples are meaningful.

Summarizing, we can conclude that in the non-depressed sample, the *Authenticity Scale* has the same three-factor structure as the original tool, which confirms Hypothesis 1.

### Internal Reliability

We examined the reliability of the *Authenticity Scale* by calculating McDonald’s omega coefficients for each subscale and for the general *Authenticity* factor. Coefficient omega uses the item factor loading and uniqueness to estimate reliability. Therefore, coefficient omega can be viewed as a more precise measure of reliability compared to coefficient alpha (Padilla and Divers, 2013). When interpreting reliability, we rely on the omega coefficients; however, we also provide Cronbach alphas for reference purposes, since all previous validation studies of the *Authenticity Scale* used alpha to estimate reliability. This will allow researchers to compare the reliability of the scale validated in different countries. Reliability measured with McDonald’s omega was satisfactory for all subscales: ω = 0.782 for the *Authentic Living* scale, ω = 0.792 for the *Accepting External Influence* scale, and ω = 0.843 for the *Self-Alienation* scale. The proportion of the second-order factor explaining the variance at the first-order factor level (hierarchical omega) was 0.893. Cronbach alpha values were 0.644, 0.708, 0.804 for the *Authentic Living*, *Accepting External Influence*, and *Self-Alienation* subscales respectively. The internal consistency of the general second-order *Authenticity* factor was also high (α = 0.838).

### Construct Validity

To evaluate the construct validity, correlations between the *Authenticity* factors and several convergent measures were examined (see Table 5). As expected, the *Authenticity Scale* components had relatively high correlations with self-esteem and depression. *Authentic Living* had positive moderate correlations with *Self-Esteem* (0.43, *p* < 0.01; medium ES) and negative one with *CES-D* scores (−0.43, *p* < 0.01; medium ES). *Accepting External Influence* and *Self-Alienation* negatively correlated with *Self-Esteem* (−0.32 and −0.55, *p* < 0.01; medium and large ES respectively) and had positive correlations with *CES-D* (0.25 and 0.52, *p* < 0.01; medium and large ES respectively). The correlations between the *Authenticity Scale* and mental wellbeing were moderate: *Authentic Living* positively correlated with *WEMWBS* (0.38, *p* < 0.01), while *Accepting External Influence* and *Self-Alienation* had negative relations to this measure (−0.22 and −0.34 respectively, *p* < 0.01); all correlations were medium in terms of ES.

**TABLE 5:** Associations among the *Authenticity Scale* and the measures of mental wellbeing, subjective wellbeing, and depression.

Measures	*Authentic Living*	*Accepting External Influence*	*Self-Alienation*	*N*	*M*	*SD*	*Score range*	*Cronbach’s alpha* (α)
*Mental wellbeing*								
*WEMWBS*	0.38**	−0.22**	−0.34**	1,298	50.30	9.27	14–70	0.89
*Subjective wellbeing*								
*SWLS*	0.37**	−0.17**	−0.35**	240	20.89	6.57	5–35	0.83
*PA*	0.20**	−0.19**	−0.26**	510	12.35	2.82	4–20	0.67
*NA*	−0.31**	0.22**	0.38**	499	6.56	3.95	5–25	0.80
*Self-Esteem*	0.45**	−0.34**	−0.55**	517	31.23	5.24	14–40	0.83
*Depression*								
*CES-D*	−0.43**	0.25**	0.52**	1,213	26.01	11.08	5–59	0.89
*N*	2,188	2,188	2,188					
*M*	13.49	13.64	12.56					
*SD*	4.24	5.19	6.10					
*Score range*	3–21	4–28	4–28					

WEMWBS, mental wellbeing; SWLS, satisfaction with life; PA, positive affect; NA, negative affect; CES-D, depression.

**−*p* < 0.01.

Furthermore, *Authentic Living* had positive correlations with *SWLS* and *PA* (0.37 and 0.20, *p* < 0.01; medium and small ES respectively), and negatively connected with *NA* (−0.31, *p* < 0.01; medium ES). Correlations between *Accepting External Influence* and *Self-Alienation* and subjective wellbeing measures were inverted: *Accepting External Influence* and *Self-Alienation* negatively and weakly correlated with *SWLS* (−0.17 and −0.35, *p* < 0.01; small and medium ES respectively) and *PA* (−0.19 and −0.26 respectively, *p* < 0.01; small ES) and positively correlated with *NA* scores (0.22 and 0.38 respectively, *p* < 0.01; small and medium ES respectively). It can be seen from Table 5 that *Self-Alienation* does indeed have higher correlations with all convergent measures, in comparison with the *Authentic Living* and *Accepting External Influence*.

Hence, we can conclude Hypothesis 2 was also confirmed.

### Descriptive Statistics

Table 6 characterizes the sample according to age and sex and presents the mean, standard deviation, effect size (Hedge *g*) on the sex and age differences and median of the *Authenticity Scale* for the total sample and subsamples. Consistent with the results of the multi-group CFA, the mean and median observed *Accepting External Influence* and *Self-Alienation* scores decrease with age, while males and females do not differ in subscale scores.

**TABLE 6:** The *Authenticity Scale* scores according to sex and age.

		Total	Sex		Age	
				
			Male	Female	Youth (18–25 y.o.)	Adult (26–70 y.o.)
*n* (%)		2188 (100%)	482 (22%)	1669 (78%)	1227 (62%)	513 (38%)
*Authentic Living*						
Effect size (Hedge *g*)		–	0.04		0.00	
	Mean (*SD*)	10.46 (4.21)	10.58 (4.40)	10.42 (4.15)	10.50 (4.03)	10.48 (4.15)
	Median [95% CI]	10 [10–11]	10 [10–11]	10 [10–11]	10 [10–11]	10 [10–11]
*Accepting External Influence*						
Effect size (Hedge *g*)		–	0.02		0.13	
	Mean (*SD*)	13.55 (5.20)	13.63 (5.32)	13.53 (5.17)	13.69 (5.27)	13.01 (5.01)
	Median [95% CI]	13 [13–14]	14 [13–14]	13 [13–14]	13 [13–14]	13 [12–14]
*Self-Alienation*						
Effect size (Hedge *g*)		–	0.04		0.51	
	Mean (*SD*)	12.58 (6.09)	12.39 (6.15)	12.64 (6.07)	13.62 (6.12)	10.60 (5.33)
	Median [95% CI]	12 [11–12]	11 [11–12]	12 [11–12]	13 [13–14]	10 [9–10]

*The median’s confidence interval has been estimated for each group through bootstrapping with 5000 replicates.*

## Discussion

The current research aimed to validate the 12-item *Authenticity Scale* (Wood et al., 2008) in Russian culture. With some changes, the *Authenticity Scale* appears to be a well-working instrument in Russia. Moreover, its psychometric properties are close to the original ones and those found in the adaptations of the scale to other, mainly European, cultures. Our hypotheses regarding structural and convergent validity were confirmed.

There were some problems caused by language nuances and shades (Kernis and Goldman, 2006). After some iterations, we succeeded in finding such language claims for the wordings, which would give good score representativeness. Compared with the original structure of the *Authenticity Scale*, there were some changes in the Russian version. Firstly, items 1, 9, and 11 initially related to the *Authentic Living* subscale were negatively reworded. Secondly, negatively reworded item 1 was moved from the *Authentic Living* to *Accepting External Influence.* In fact, the original item 1 (“I believe that it is better to be yourself than to be popular”) involved the opposition of two entities (to be yourself and to be popular) that can coexist together. When reversing this composite item, the emphasis was made on” to be popular,” which is why the item fell into the second subscale. If it were initially formulated more unambiguously (for instance, “I believe that it is important, to be yourself”), then when reverted it would sound like “I don’t believe that it is important, to be yourself”), and then, perhaps, would remain in the first subscale. As a result, *Authentic Living* was loaded with only three items. Thirdly, in order to make the model more parsimonious, item 4 was dropped from the *Accepting External Influence* subscale due to its high residual variance and the high residual covariance with the item 5. Due to these corrections, we kept the three-factor initial model of the *Authenticity Scale* but it included 11 items only vs. the original scale. Perhaps this problem occurred due to the cultural specifics of Russians (Marcinkovskaya, 1994) who cannot admit that they could sometimes behave inauthentically or not be authentic individuals, which resulted in a high skewedness score of *Authentic Living* subscale. To be oneself is a national value for Russians, and they try to be at all costs. Since most of the authors in presenting descriptive statistics are limited only to the mean score and standard deviation indicators, we were not able to compare our data with those obtained by other researchers. However, Grijak (2017) also reports very high rates of asymmetry across all scales.

Despite some problems caused by editing the wording of the scale, the Russian version of the *Authenticity Scale* has acceptable scores of internal consistency, construct reliability, and is characterized by stable reproducibility of measurement results in different samples. Both EFA and CFA indicated that *Authenticity Scale* has kept the original three-factor structure and comprises *Authentic Living*, not *Accepting External Influence*, and a lack of *Self-Alienation*. After having compared several alternative solutions (unidimensional, hierarchical, three-factor correlated, and bi-factor), the best fit was received for the oblique three-factor model with the hierarchical solution (with general *Authenticity* as the higher-order factor and *Authentic Living, Accepting External Influence*, and *Self-Alienation* representing first-order latent factors).

Furthermore, as in some previous research (Wenzel and Lucas-Thompson, 2012; Satici et al., 2013), we found no sex differences in the authenticity scores. Despite Russia being a traditional culture without gender equality, Russian women have had many social and economic rights over the last centuries and were respected, which might result in their opportunities to be oneself. Moreover, this fact is in line with the so-called gender equality paradox, according to which in those cultures where no equality is, gender differences in psychological qualities probably can be less pronounced (Kaiser, 2019).

As for age differences, it was found that *Accepting External Influence* and *Self-Alienation* scores are much higher in youth compared to adults. This fact is not consistent with Rogers’ (1961) idea that people lose their authenticity with age. To understand this, we have to take into account some features of the contemporary reality in which Russian emerging adults face many challenges and stresses and need to have attachments and figures of admirations that, on the one hand, provide support to the youth but, on the other hand, limit their freedom by rules and expectations regarding their future (Robinson et al., 2013, 2016). In Russia, they have one more source of stress, namely, conscription that deprives young men of some of the freedom needed for their authentic living and strengthens emotional and social tension in their families. The comparatively higher tendency of young people to self-alienation might be because of the interplay between identity synthesis and identity confusion during emerging adulthood. Many young people are not ready to accept a wide range of conditions for exploring alternative gender, religious or political identities offered by modern society. As a result, they have a disintegrated sense of self and are “stuck” in the identity development process due to a fear of making the wrong choice, which, in turn, damages their authenticity (Luyckx et al., 2008).

Although the *Authenticity Scale* is used in some studies for investigating people with anxiety and depression (e.g., Blomgren and Strååht, 2018), as far as we know, the factor structure in populations with different levels of clinical depression have not been examined before. In our study, the item intercepts the *Authenticity Scale* are not similar for people with high and low levels of depressive symptoms. Hence, the results of the item scores comparison on the *Authenticity Scale* in people with different levels of depression should be interpreted with caution and additionally checked using alternative measures.

We have also checked the convergent validity of the *Authenticity Scale* using the same questionnaires as in other cultural adaptations. According to our expectations, we have revealed precise, easily interpreted results: in line with İlhan and Özdemir (2013); Di Fabio (2014), Grégoir et al. (2014); Bryan et al. (2015), Vainio and Daukantaitė; (2015); Grijak (2017), and Blomgren and Strååht (2018), the *Authentic Living* subscale was really positively connected with mental wellbeing, positive affect, self-esteem and life satisfaction, negatively correlated with negative affect and depressive symptoms whereas *Accepting External Influence* and *Self-Alienation* formed inverse connections. Noteworthy, the most robust links with well/ill-being indicators were formed by the *Self-Alienation* subscale, demonstrating that it is more important not to be self-alienated than it is to be authentic. This fact also is interpreted as, in line with Baumeister et al. (2001), “bad is stronger than good,” and psychological phenomena are more differentiated, nuanced, and impressive on the negative vs. positive pole of the *Authenticity Scale*. It is easy to understand, considering the adaptive function of all traits (McAdams and Pals, 2006), including narrow ones: whereas negative features are a threat to survival, the lack of positive ones is entirely consistent with the existence of a low level of stability, just without peak experiences.

Altogether, although this work resulted in a modified version of the *Authenticity scale*, rather than an exact adaptation, this tool does work. As for the need for changes, in our opinion, they can be explained by the cultural factor. Whereas the scale was developed in the individualistic culture, Russia is situated between the West and East; it is moderately collectivistic (Hofstede et al., 2010), which might result in the slightly different structure of the scale. Furthermore, as a survival culture (World Values Survey, 2020), Russia seems to focus on the pragmatic short-term perspective, which also may make authenticity not a very high-demand feature. At the same time, Russia has a long tradition of art and humanities and, generally, humanistic educational techniques that gave support for self-respect and authenticity development.

However, the outcomes obtained give evidence for considering the *Authenticity Scale* valid and reliable tool that may be in demand of many scholars and practitioners.

## Limitations and Prospects

Even though we have got the evidence of validity and reproducibility of results measured by the *Authenticity Scale*, the study has several limitations. One of them is the relative homogeneity of the sample: the results obtained are more relevant for female university students. Therefore, future research has to study the generalizability of measurement results on the more balanced population samples.

One more limitation is due to the need to slightly modify the structure of the initial questionnaire, which made cross-cultural comparison on the *Authenticity Scale* doubtful. Thus, for cross-cultural research tasks, it will probably be necessary to go back to the original version of the statements, despite its weakness compared to the modified tool. In the future, we should check several more versions again to find the optimal one that works well in Russia and is suitable for cross-cultural comparisons.

Finally, in the current study, a degree to which individual test scores of the *Authenticity Scale* keep stable at a different point in time hasn’t been estimated. As a line of further investigation of the *Authenticity Scale*, we plan to measure test–retest reliability.

Despite these shortcomings, the resulting version of the *Authenticity Scale* can be used in studies of positive and higher personality phenomena, meaningful or critical events, life-calling, and self-actualization. In addition, our results seem to have applications in evaluating the efficiency of various educational and psychotherapeutic techniques, and identifying the risk and sub-clinical population groups. Finally, they could become the basis for the personal training programs.

## Conclusion

In the current paper, we have summarized results of the validation study aimed at the adaptation of the *Authenticity Scale* by Wood et al. (2008) in Russia. The scale was undergone the standard procedure of adaptation of the questionnaire to the other culture – translation, back-translation, the examination of internal consistency, age trends and sex differences, structural and convergent validity. As a result, we have revealed a modified version of the tool, with a slightly different structure. At the same time, this structure was kept across different samples, which gives substantial evidence for the stability of this version in the Russian culture. Thus, the method can be recommended for use in research and practice.

## Data Availability Statement

The raw data supporting the conclusions of this article will be made available by the authors, without undue reservation.

## Ethics Statement

The studies involving human participants were reviewed and approved by the National Research University Higher School of Economics Committee on Inter-university Surveys and Ethical Assessment of Empirical Research. Written informed consent to participate in this study was provided by the participants, and where necessary, the participants’ legal guardian/next of kin.

## Author Contributions

SN-B developed the main idea of the manuscript, collected the data, organized the database, wrote the first draft of the manuscript, contributed to the manuscript revision, read, and approved the submitted version. SR contributed to the study’s conception and design, performed the statistical analysis, and contributed to the manuscript revision. JM supervised all the research, contributed to the design of the study and the model specifications, provided the feedback, and rewrote sections of the manuscript. All the authors approved the submitted version of the manuscript.

## Conflict of Interest

The authors declare that the research was conducted in the absence of any commercial or financial relationships that could be construed as a potential conflict of interest.

## Appendix

The *Authenticity Scale*: Original, Russian, and corresponding English versions.

****

### Instruction

Please read the list of statements provided and rate them in terms of how they characterize your habits and behavior. Please tick the answer that best describes you.

**Table:**
