# Corrigendum: The Authenticity Scale: Validation in Russian Culture

**DOI:** 10.3389/fpsyg.2021.675919

**Published:** 2021-04-20

**Authors:** Sofya Nartova-Bochaver, Sofia Reznichenko, John Maltby

**Affiliations:** ^1^School of Psychology, National Research University Higher School of Economics, Moscow, Russia; ^2^College of Medicine, Biological Sciences and Psychology, University of Leicester, Leicester, United Kingdom

**Keywords:** Authenticity Scale, wellbeing, validation, reliability, Russian culture

In the original article, there was a mistake in Figure 3 “The optimal CFA model tested for the Authenticity Scale compared with the original model (Wood et al., 2008).” as published. Near the item 1, which is included in Acceptance External Influence subscale, the sign “–” was mistakenly put. It should be removed. The corrected Figure 3 “The optimal CFA model tested for the Authenticity Scale compared with the original model (Wood et al., 2008)” appears below.

**Figure 3:**
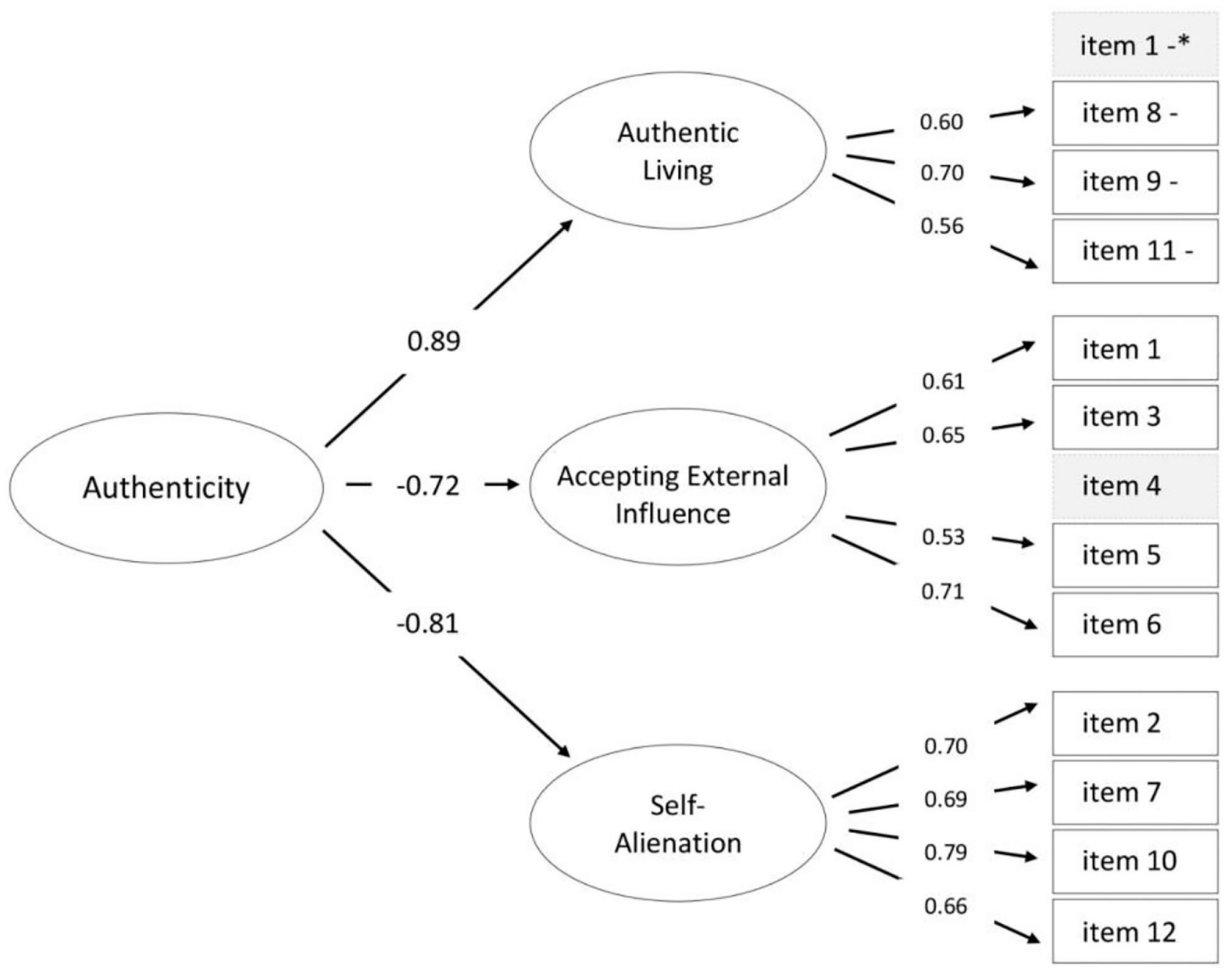
The optimal CFA model tested for the *Authenticity Scale* compared with the original model (Wood et al., 2008). Blocks dotted gray indicate excluded items. ^*^ – item one was included in *Accepting External Influence*. “–” indicates negatively phrased items. Error variances omitted for clarity.

In the original article, there was also a mistake in the Appendix table “The Authenticity Scale: Original, Russian, and Corresponding English Versions” as published. The text of Item 1 (both English and Russian Wording) was incorrectly highlighted in bold. The text should be in normal (regular) style. The corrected Appendix table “The Authenticity Scale: Original, Russian, and Corresponding English Versions” appears below.

The authors apologize for these errors and state that they do not change the scientific conclusions of the article in any way. The original article has been updated.

**Appendix**

The *Authenticity Scale*: Original, Russian, and corresponding English versions.

**Инструкция**:

Пожалуйста, прочтите список приведенных утверждений и оцените их с точки зрения того, насколько они характеризуют Ваши привычки и поведение. Поставьте галочку в ячейке под тем ответом, который подходит Вам.

**Instruction:**

Please read the list of statements provided and rate them in terms of how they characterize your habits and behavior. Please tick the answer that best describes you.

## Appendix

**Table:**

**Item code**	**The original *Authenticity Scale* (Wood et al., 2008)**	**The Russian version of the *Authenticity Scale* English wording**	**The Russian version of the *Authenticity Scale* Russian wording**
1	I think it is better to be yourself than to be popular (AL)	I think it is better to be popular than to be yourself (AEI)	Для меня важнее понравиться другим, чем «оставаться самим собой (ПВВ)
2	I don't know how I really feel inside (SA)	I don't know how I really feel inside (SA)	Я не знаю, что я чувствую на самом деле (СО)
3	I am strongly influenced by the opinions of others (AEI)	I am strongly influenced by the opinions of others (AEI)	Мои поступки и взгляды меняются в зависимости от мнения других (ПВВ)
4	I usually do what other people tell me to do (AEI)	I usually do what other people tell me to do (*Deleted*)	Обычно я делаю то, о чем меня просят другие (*Deleted*)
5	I always feel I need to do what others expect me to do (AEI)	I always feel I need to do what others expect me to do (AEI)	Я считаю, что должен делать то, чего от меня ждут другие (ПВВ)
6	Other people influence me greatly (AEI)	Other people influence me greatly (AEI)	Окружающие очень сильно влияют на меня (ПВВ)
7	I feel as if I don't know myself very well (SA)	I feel as if I don't know myself very well (SA)	Мне кажется, что я не знаю себя достаточно хорошо (СО)
8	I always stand by what I believe in (AL)	**I do not always succeed in upholding what I believe in** (AL)	**Мне не всегд****а** **уд****а****ется отстоять то, во что я верю **(АЖ)
9	I am true to myself in most situations (AL)	**I can't say that I am true to myself in most situations** (AL)	**Не могу ск****а****з****а****ть, что я всегд****а** **быв****а****ю верен себе **(АЖ)
10	I feel out of touch with the “real me” (SA)	I feel out of touch with the “real me” (SA)	Я чувствую, что я не в контакте с «истинным Я» (СО)
11	I live in accordance with my values and beliefs (AL)	**I don't live in accordance with my values and beliefs** (AL)	**Мне трудно жить в соответствии с моими ценностями убеждениями **(АЖ)
12	I feel alienated from myself (SA)	I feel alienated from myself (SA)	Я кажусь незнакомцем самому себе (СО)

*Reverted items are in bold. Deleted – item 4 was excluded during the CFA. The items' belongings to each scale is indicated in the brackets: AL, Authentic Living (АЖ – Аутентичная жизнь); AEI, Accepting External Influence (ПВВ – Принятие внешнего влияния); SA, Self-Alienation (СО – Самоотчуждение)*.

*Responses were made on a seven-point scale: from 1 (не относится ко мне вообще; does not describe me at all) to 7 (полностью относится ко мне; describes me very well)*.
